# Neuro-ophthalmic Manifestations of Coronavirus Disease 2019 and Its Vaccination: A Narrative Review

**DOI:** 10.18502/jovr.v18i1.12731

**Published:** 2023-02-21

**Authors:** Mohadeseh Feizi, Danielle R. Isen, Mehdi Tavakoli

**Affiliations:** ^1^Ophthalmic Research Center, Research Institute for Ophthalmology and Vision Science, Shahid Beheshti University of Medical Sciences, Tehran, Iran; ^2^University of Alabama at Birmingham Heersink School of Medicine, Department of Ophthalmology and Visual Sciences, Birmingham, Alabama, USA

**Keywords:** Corona Virus, COVID-19, Neuro-ophthalmology, Vaccination, Vision Loss

## Abstract

Coronavirus disease 2019 (COVID-19) is a current pandemic caused by SARS-CoV-2 that has vastly affected the whole world. Although respiratory disease is the most common manifestation of COVID-19, the virus can affect multiple organs. Neurotropic aspects of the virus are increasingly unfolding, in so far as some respiratory failures are attributed to brainstem involvement. The neuro-ophthalmic manifestations of COVID-19 and the neuro-ophthalmic side effects of vaccination were reviewed. The major findings are that the SARS-CoV-2 infection commonly causes headaches and ocular pain. It can affect the afferent and efferent visual pathways by ischemic or inflammatory mechanisms. Optic nerve may be the origin of transient or permanent visual loss from papillophlebitis, idiopathic intracranial hypertension, or optic neuritis. Cerebrovascular strokes are not uncommon and may lead to cortical visual impairment or optic nerve infarction. SARS-CoV-2 may affect the pupillomotor pathways, resulting in tonic pupil (Adie's syndrome) or Horner's syndrome. Cranial neuropathies including third, fourth, sixth, and seventh nerve palsies have all been reported. Rhino-orbital mucormycosis superinfections in COVID-19 patients receiving steroids or other immunosuppressive therapies may result in unilateral or bilateral visual loss and ophthalmoplegia. Autoimmune conditions such as Guillain-Barré, Miller-Fisher syndrome, and ocular myasthenia have been reported.

##  INTRODUCTION

Coronavirus disease-2019 (COVID-19) is a novel β coronavirus of group 2B, first reported in December 2019 in Wuhan city, that rapidly disseminated all over the world, resulting in an immense pandemic.^[1]^ The disease presents in a widely variable range from asymptomatic carriers or mild infection to severe acute respiratory syndrome (SARS), multi-organ failure, and death. Besides respiratory distress syndrome, SARS-CoV-2 may affect many other organs and cause cardiac or renal injury, dermatologic conditions, gastrointestinal symptoms, coagulopathy, severe inflammatory reaction, and central or peripheral nervous system involvement.^[2]^ Up to one-third of patients with COVID-19 have neurological complications, and the incidence appears to be higher in patients with more severe infections.^[3]^ The most common severe neurologic manifestations of COVID-19 are acute cerebrovascular disease and altered level of consciousness.^[4]^ A retrospective study in Wuhan reported neurological symptoms in 36.4% of the hospitalized patients.^[1]^ Neurologic manifestations are mainly reported to be associated with severe cases of COVID-19.^[1,5]^ Moreover, COVID-19-related cases of ischemic stroke involving visual pathways have resulted in prolonged hospitalizations and fatality.^[6,7]^ Findings have suggested that ischemic strokes in patients with COVID-19 disease are more severe and disabling than strokes in the uninfected subjects.^[8]^ In contrast, isolated cranial neuropathies involving the eye are typically associated with mild to moderate COVID-19 disease that improves spontaneously or with local protocols.^[9,10]^

A recent review reported that over 4% of patients with COVID-19 had ophthalmic findings requiring clinical attention.^[11]^ Based on an initial study in Wuhan, visual impairment was described in 3 out of 214 (1.4%) hospitalized patients with COVID-19.^[1]^

Various neuro-ophthalmologic manifestations have been reported in patients with COVID-19 infection. In this article, we reviewed and categorized SARS-CoV-2-related neuro-ophthalmic presentations.

### Mechanisms of Neurologic Involvement in COVID-19

Several mechanisms have been postulated for neurologic involvement in COVID-19 disease. Direct viral invasion, hypoxia, hypercoagulable state, inflammatory reactions related to cytokine storm, delayed autoantibody formation, endothelial dysfunction, and retrograde axonal transport of the infection via cranial and peripheral nerves (most notably, the olfactory nerve) are the proposed underlying mechanisms for neuro-ophthalmic manifestations of COVID-19.^[2,5]^ It is thought that direct viral invasion is mediated by viral fusion to the angiotensin-converting-enzyme 2 (ACE-2) receptor, which is expressed on the surface of pulmonary type II alveolar epithelial cells as well as neurons and glial cells.^[12]^ The SARS-COV-2 virus can affect nearly all neuro-ophthalmic pathways [Table 1]. COVID-19 can cause a wide range of pathologies within the afferent and efferent visual pathways. SARS-CoV-2 can cause central nervous system conditions such as seizures,^[13]^ anosmia or ageusia,^[14]^ altered level of consciousness, posterior reversible encephalopathy syndrome (PRES),^[15,16]^ neuromyelitis optica (NMO) spectrum disorder,^[4,17]^ myelin oligodendrocyte glycoprotein (MOG)-associated disease,^[3,18,19,20,21,22]^ acute disseminated encephalomyelitis (ADEM),^[23]^ cerebral venous sinus thrombosis (CVST),^[24,25]^ and cerebrovascular strokes.^[2][6,7][8]^ It can also affect the peripheral nervous system, leading to conditions such as Guillain-Barré,^[26]^ Miller Fisher syndrome,^[27,28]^ polyneuritis cranialis,^[28]^ and myasthenia gravis.^[29,30]^

SARS-CoV-2 is associated with pathologies of the afferent visual pathways including optic neuritis (idiopathic^[5,14,31,32,33]^ or immune-mediated),^[3][4,17][18][19][20][21][22]^ optic nerve infarction,^[34]^ papillophlebitis,^[35]^ and idiopathic intracranial hypertension.^[36,37,38]^ The efferent visual pathways can be lesioned by COVID-19-related isolated cranial neuropathies,^[9,27,39,40,41]^ nystagmus,^[42,43,44,45,46]^ tonic pupil,^[47,48,49,50,51]^ Horner's syndrome,^[12,13,52]^ and opsoclonus-myoclonus-ataxia syndrome (OMAS).^[53,54,55]^ Mucormycosis infections,^[56]^ which are of exponentially increasing incidence in COVID-19 patients, can occur as a superinfection and result in lesions of the afferent or efferent visual pathways or even both.^[57,58]^

### Interval Between Infection and Neuro-Ophthalmic Manifestations

Neuro-ophthalmic manifestations of COVID-19 may present either concurring with systemic and pulmonary symptoms or days to several weeks after their resolution. Timeline latency between acute viral symptoms and neuro-ophthalmic manifestation, auto-antibody formation, and good response to steroid therapy favor an immune-mediated mechanism.^[49]^ Guillain-Barré, Miller Fisher syndrome, NMO, MOG-associated disease, ADEM, tonic pupil, rhombencephalitis, Bickerstaff encephalitis, myasthenia gravis (MG), and OMAS were all postulated to have delayed-immune mediated mechanisms related to COVID-19. The delayed immune response may be from antibodies directed against SARS-CoV-2 proteins that cross-react with cellular proteins through a mechanism known as molecular mimicry. Alternatively, COVID-19 infection may impair immunologic self-tolerance.^[29]^

### Headache/Ocular Pain

Ocular pain and headache are common and may be the initial manifestation of COVID-19 infection.^[1,11,59]^ Headache was reported in 71% and ocular pain in 34% by healthcare workers in the Netherlands in patients with a positive SARS-CoV-2 polymerase chain reaction (PCR).^[60]^ In another study, eye pain was amongst the most common of the ocular symptoms and seen in 16% of patients.^[61]^ Photophobia and itchy eyes were seen at a similar frequency (18% and 17%, respectively).

Headache characteristics in COVID ‐ 19 patients were moderate to severe, pulsating or pressing, bilateral, and mostly in the temporo-parietal, forehead, or periorbital regions. It has been postulated that an increase in pro-inflammatory cytokines, hypoxia, or activation of the trigeminal nerve endings from direct viral insult or vasculopathy explain the underlying pathophysiology of headache in COVID-19 disease.^[62]^ However, the exact mechanism is not clear, and viral encephalitis and meningitis should be considered.

Overall, it seems that headache and periocular pain are the most prevalent neuro-ophthalmic presentations of COVID-19 but more comprehensive epidemiological studies are required to evaluate the prevalence of each neurological symptom.

### Optic Neuritis and Other Optic Nerve Disorders

Optic neuritis has been reported in association with COVID-19 infections in multiple cases where no other etiologies have been determined.^[5,14,31,32,33]^ COVID-19 has also been the harbinger to optic neuritis in NMO spectrum disorder^[4,17]^ and MOG-related disease.^[3,18,19,21,63,64]^ Optic neuritis in association with COVID-19 infection has also been reported in the pediatric age group even in the context of demyelinating conditions.^[65]^ COVID-19-related optic neuritis had a good response to intravenous (IV) corticosteroid therapy in all cases except four.^[5,14,22,32]^

Optic neuritis was reported in conjunction with panuveitis in one case of COVID-19, which resulted in optic atrophy despite corticosteroid treatment. However, the patient only received oral prednisone without high-dose IV corticosteroids. The authors postulated that ischemic insult due to the prothrombotic capacity of COVID-19 or an inflammatory reaction resulted in optic atrophy in this case.^[32]^

Novi et al reported an adult patient with COVID-19 disease and bilateral retrobulbar optic neuritis with enhancing lesions in optic nerves, brain, and thoracic spine, consistent with ADEM.^[23]^

Cerebrovascular ischemia is a known complication of SARS-CoV-2 infection.^[1]^ Optic nerve infarction due to internal carotid artery occlusion was reported in a 50-year-old COVID-positive male by Tavakoli et al.^[34]^ Central retinal artery occlusion^[66]^ or ophthalmic artery occlusion^[67]^ due to internal carotid artery stroke have also been reported in COVID-19 disease.

There is one report of papillophlebitis in a 40-year-old male that occurred four weeks after the resolution of a typical COVID-19 infection with elevated D-dimer and fibrinogen levels. The authors proposed that the COVID-19 cytokine storm induced a hypercoagulable state and thrombotic microangiopathy, increasing the risk of papillophlebitis.^[35]^

Idiopathic intracranial hypertension can occur in adults with COVID-19 disease, but it is rare, and most of the cases have been reported in children.^[36,37,38]^ Two of these cases were associated with increased intracranial pressure inducing sixth nerve palsies in idiopathic intracranial hypertension due to COVID-19 multisystem inflammatory syndrome in children (MIS-C).^[36,37]^

### Pupillary Abnormalities

Tonic (Adie's) pupil refers to parasympathetic denervation of the pupil sphincter, which causes poor constriction of the pupil to the light but the reaction to the accommodation remains relatively spared. Tonic pupil has been reported in association with COVID-19, including unilateral^[47]^ and bilateral^[48]^ cases. One patient with COVID-19-related bilateral tonic pupil also had inflammatory multifocal choroiditis.^[68]^ Additionally, Adie-Holmes syndrome (with additional finding of loss of the deep tendon reflexes) has been described in a patient with COVID-19 infection.^[69]^ Lastly, Ordás et al reported a case of tonic pupil with contralateral trochlear palsy in association with COVID-19 infection.^[49]^

Tonic pupil is often idiopathic, but it has been associated with other infections (e.g., syphilis, Lyme's disease, influenza or herpes viruses), autoimmune processes, trauma, choroidal and orbital tumors, and surgery.^[47]^ In each of the above cases of tonic pupil,^[47]^ there was a delay (ranging from two days to one month) between the onset of COVID-related respiratory symptoms and development of tonic pupil. Most of the authors hypothesized that tonic pupil in COVID-19 is secondary to a post-viral delayed immune-mediated injury rather than direct viral entry into the central nervous system. Moreover, a taper of prednisolone has been utilized for treatment with subsequent improvement in the tonic pupil.^[50]^

Horner's syndrome has also been rarely described in COVID-19 disease.^[12,13,52]^ In each case, COVID-19 pneumonia involving the upper part of the lungs was the suggested etiology of Horner's syndrome, and no other causes were discovered with subsequent imaging studies.

### Central Visual Impairment and Visual Field Defects

COVID-19 disease can induce hypercoagulable and inflammatory states, increasing the risk of cerebrovascular stroke.^[6,70]^ There are reports of ischemic stroke involving the visual pathways in association with SARS-CoV-2 infection, resulting in homonymous field defects^[2]^ or even cortical blindness from bilateral occipital infarcts.^[7]^ Bondira et al reported a patient with COVID-19 disease and bilateral occipital strokes, resulting in a partial right homonymous hemianopsia and difficulty with reading.^[6]^ Priftis et al presented a COVID-positive patient with a left occipito-temporal ischemic stroke, resulting in alexia without agraphia syndrome and right homonymous hemianopsia.^[8]^

COVID-induced PRES has been reported, resulting in transient cortical visual loss^[15]^ or even hallucinatory palinopsia.^[16]^ COVID-associated CVST has also been described but is uncommon, and only a few cases have been associated with blurry vision and papilledema.^[24,25]^ The disproportionally low frequency of reported visual involvement in COVID-associated CVST may be because of the severity of other symptoms or an altered level of consciousness.

In addition, there are reports of visual loss due to pituitary apoplexy in the context of acute COVID-19 infection.^[71,72]^

**Table 1:** A summary of reported neuro-ophthalmic manifestations of COVID-19.


orange**Clinical entity**	orange**Clinical characteristics**	orange**References**
**Headache and ocular pain**	Common and may be the initial manifestations. Moderate to severe, pulsating/pressing, bilateral, temporo-parietal, forehead, or periorbital regions.	Mao L et al^[1]^, 2020; Chwalisz BK et al, 2020^[11]^; Huang C et al^[59]^, 2019
**Optic neuritis**	Unilateral or bilateral, may be associated with neuromyelitis optica (NMO) spectrum disorders, myelin oligodendrocyte (MOG)-related disease, panuveitis, acute disseminated encephalomyelitis (ADEM).	Caudill GB et al ^[31]^, 2020; Benito-Pascual B et al^[32]^, 2019; Marcos Aet al^[5]^, 2020; Rodríguez-Rodríguez MS et al^[14]^, 2021; Deane K et al^[33]^, 2021; Novi G et al^[23]^, 2020
**Optic nerve infarction**	Vision loss due to internal carotid artery occlusion, optic nerve ischemia revealed on DWI sequence.	Tavakoli et al^[34]^, 2019
**Papillophlebitis**	Decreased visual field sensitivity, dilated and tortuous retinal vessels, disc edema, and retinal hemorrhage; decreased vision due to macular edema.	Insausti-García A et al^[35]^, 2020
**Idiopathic intracranial hypertension**	More reported in children than adults due to multisystem inflammatory syndrome (MIS).	Verkuil LD et al^[36]^, 2020; Sofuoğlu AI et al^[37]^, 2021; Khalid MF et al^[38]^, 2021
**Tonic Adie's pupil**	Unilateral or bilateral, associated with multifocal choroiditis; trochlea palsy.	Gopal M et al^[47]^, 2021; Quijano-Nieto BA et al^[48]^, 2021; Ortiz-Seller A et al^[68]^, 2020; Kaya Tutar N et al^[69]^, 2021; Ordás CM et al^[49]^, 2020
**Horner syndrome**	Associated with pneumonia involving the upper part of the lung.	Popiołek A et al^[52]^, 2021; Naor MS et al^[12]^, 2021; Portela-Sánchez S et al^[13]^, 2021
**Visual field defect and central visual impairment**	Cerebrovascular stroke resulting in homonymous visual field defect, cortical visual blindness, reading difficulties.	Tisdale AK et al^[70]^, 2020; Bondira IP et al^[6]^, 2021; Cyr DG et al^[7]^, 2020; Priftis K et al^[8]^, 2021
**Posterior reversible encephalopathy syndrome (PRES)**	Transient cortical visual loss and hallucinatory palinopsia.	Kaya Y et al^[15]^, 2020; Ghosh R et al^[16]^, 2020
**Cranial nerve palsy**	Isolated or multiple cranial nerve involvement including third, fourth, sixth, and seventh. Can occur in the context of Miller Fisher syndrome, Guillain-Barré, myasthenia gravis, venous sinus thrombosis, and increased intracranial pressure.	Ordás CM et al^[49]^, 2020; Douedi S et al^[39]^, 2021; Cicalese MP et al^[73]^, 2022; John C et al^[40]^, 2020; Greer CE et al^[9]^, 2020; Dinkin M et al^[27]^, 2020; de Oliveira R de MC et al^[74]^, 2020; Gutiérrez-Ortiz C et al^[28]^, 2020; Sansone P et al^[26]^, 2021; Restivo DA et al^[29]^, 2020; Mas Maresma L et al^[30]^, 2020; Lima MA et al^[10]^, 2020; Juliao Caamaño DS et al^[75]^, 2020
**Nystagmus and abnormal ocular movement **	Acquired nystagmus due to acute labyrinthitis, benign paroxysmal positional vertigo, rhombencephalitis, Bickerstaff encephalitis, opsoclonus-myoclonus-ataxia syndrome (OMAS).	Perret M et al^[42]^, 2021; Picciotti PM et al^[43]^, 2021; Wong P et al^[44]^, 2022; Llorente Ayuso L et al^[45]^, 2021; Nelson JL et al^[53]^, 2022; Emamikhah M et al^[54]^, 2021
**Rhino-orbital-cerebral mucormycosis (ROCM)**	Mostly occurs in patients receiving high-dose corticosteroid; presented with peri-ocular edema, vision loss, ptosis, and ophthalmo-paresis.	Sen M et al^[57]^, 2021; Pakdel F et al^[58]^, 2022
	
	

### Cranial Nerve Palsies and Double Vision

Damage to third, fourth, sixth, and seventh cranial nerves have all occurred in timeline relation to COVID-19 infection. Cranial nerve involvement may develop as a primary and isolated insult or in the context of a more generalized condition such as Miller Fisher syndrome, Guillain-Barré, myasthenia gravis, venous sinus thrombosis, and increased intracranial pressure.

Isolated oculomotor palsy with and without pupillary involvement has been reported in association with COVID-19.^[39,40,73]^ Similarly, isolated abducens palsy has been described in COVID-19.^[9,27]^ In these cases, there was no enhancement of the abducens nerve pathway on MRI, and patients had underlying systemic hypertension. Such findings raised the hypothesis that uncontrolled hypertension in the acute viral illness may cause abducens palsy. An alternative theory was that the abducens palsies occurred from the leptomeningeal invasion of SARS-CoV-2, given that two of these cases had optic nerve sheath enhancement on MRI. As previously mentioned, there is one case of unilateral trochlear nerve palsy with contralateral tonic pupil^[49]^ and another with bilateral trochlear nerve palsy in the setting of COVID-related cerebral vasculitis.^[74]^

Different medications for COVID-19-related cranial neuropathies such as hydroxychloroquine, azithromycin, corticosteroids, and IV immunoglobulin have been used. However, it is not known whether the neuro-ophthalmic benefit was conferred.

Cranial neuropathy in relation to COVID-19 has also been seen in the context of Miller Fisher syndrome, Guillain-Barré, and polyneuritis cranialis. Dinkin et al described a case of presumed Miller Fisher syndrome in a COVID-positive patient with a partial left pupil involving oculomotor nerve palsy, bilateral abducens palsy, hyporeflexia, and gait ataxia.^[27]^ The deficits partially improved following IV immunoglobulin infusions. Gutiérrez-Ortiz et al detailed a similar case of Miller Fisher syndrome in a patient with right fascicular oculomotor palsy, right internuclear ophthalmoparesis, ataxia, areflexia, albuminocytologic dissociation, anosmia, ageusia, and positive reverse transcriptase-PCR for SARS-CoV-2. Most of the deficits were resolved following IV immunoglobulin infusions. These authors described a second COVID-positive case with bilateral abducens palsy, areflexia, and albuminocytologic dissociation without ataxia or limb weakness secondary to polyneuritis cranialis.^[28]^

In a systematic review published by Sansone et al, of the 41 patients with SARS-CoV-2-related Guillain-Barré syndrome, 9 (22%) had diplopia, 7 (17%) had ocular palsy, and 13 (32%) had facial palsy. The average time between the onset of SARS-CoV-2 symptoms and the first neurological signs of Guillain-Barré syndrome was 10 days.^[26]^

COVID-19 has also been the harbinger of new diagnoses of myasthenia gravis. Restivo et al detailed three patients who developed diplopia, ptosis, or dysphagia and tested positive for elevated serum acetylcholine receptor antibodies following the SARS-CoV-2 infection.^[29]^ Fatigability and other myasthenic symptoms started four to seven days after the initial COVID-19 manifestations.^[29,30]^ Similarity between SARS-CoV-2 epitopes and neuromuscular junction components is postulated to induce molecular mimicry, resulting in auto-antibody formation and subsequently post-COVID-19 myasthenia gravis.^[29]^ Isolated peripheral facial nerve palsy has also been reported either during or at the onset of the SARS-CoV-2 clinical course. In one of the eight peripheral facial nerve palsy cases reported by Lima et al, ipsilateral abducens palsy was also present.^[10]^ Facial diplegia has also been reported as a parainfectious complication of COVID-19 disease and was considered a rare variant of Guillain-Barré syndrome in the setting of concomitant albuminocytologic dissociation.^[75]^

### Abnormal Ocular Movements and Nystagmus

Acquired nystagmus has been seen in association with COVID-19,^[76]^ and may be due to various underlying etiologies including acute labyrinthitis,^[42]^ benign paroxysmal positional vertigo,^[43]^ rhombencephalitis,^[44]^ and Bickerstaff encephalitis.^[45]^

Several cases of OMAS have been described in COVID-positive patients.^[53,54,55]^ In all patients who were tested, work-up with a paraneoplastic panel was unrevealing for other causes of OMAS. A variety of infections besides SARS-CoV-2 including human immunodeficiency virus (HIV), West Nile virus, Epstein Barr virus, and enterovirus have also been associated with OMAS.^[53]^ Treatments used for COVID-associated OMAS include various combinations of the following: IV immunoglobulin, levetiracetam, sodium valproate, clonazepam, and corticosteroids. All cases were associated with a good therapeutic response and partial to complete recovery. Emamikhah et al stated that the dramatic effect of immunotherapy on recovery suggests an immune-mediated parainfectious mechanism for COVID-related OMAS.^[54]^

### Neuro-Ophthalmic Presentation Due to Secondary Mucormycosis

There is strong evidence that infection with SARS-CoV-2 increases the risk of secondary fungal infections, notably rhino-orbital-cerebral mucormycosis (ROCM). The incidence of ROCM increased to epidemic proportions during the second wave of the COVID-19 pandemic in India.^[57]^ A retrospective, observational study was conducted in India involving 2826 patients with COVID-associated ROCM. The most common primary signs included periocular/facial edema (33%), loss of vision (21%), ptosis (12%), and proptosis (11%). Ocular movement restriction and diplopia were infrequent signs (3%). Most (87%) of the patients had received systemic corticosteroids prior to developing COVID-associated ROCM, and it was the most common risk factor for the condition followed by diabetes mellitus.

### Neuro-Ophthalmic Complications of COVID-19 Vaccines

There are several reports of neuro-ophthalmic complications following COVID-19 vaccination.^[77,78,79]^ These complications occurred after all types of vaccines. They include acute ischemic stroke, intracranial hemorrhages, and cerebral venous sinus thrombosis.^[78,80]^ Other reported neuro-ophthalmic complications include: bilateral arteritic anterior ischemic optic neuropathy (AAION),^[81]^ post-vaccination cranial neuropathy, that is, third^[73]^ and sixth^[82,83,84]^ nerve palsies, facial palsy,^[84,85,86,87]^ multiple cranial nerve palsies,^[88]^ pupillary abnormalities such as Horner's syndrome, Holmes-Adie pupil, miosis and mydriasis,^[78]^ eight nerve involvement and benign paroxysmal positional vertigo,^[89]^ and post-vaccination optic neuritis.^[90,91,92]^ In multiple sclerosis patients, BNT162b2 mRNA vaccine was reported to be associated with a transient increase of MS symptoms in 3.8% but none of them needed treatment.^[93]^

##  SUMMARY

Coronavirus Disease 2019 and also all types of vaccines that have been made against it can affect the afferent and efferent visual pathways by ischemic or inflammatory mechanisms. The optic nerve may be the origin of transient or permanent visual loss from papillophlebitis, idiopathic intracranial hypertension, or optic neuritis. Cerebrovascular strokes are not uncommon and may lead to cortical visual impairment. The pupillomotor pathways and cranial nerve including the third, fourth, sixth, and seventh nerve may also be involved. Early detection of the neuro-ophthalmic features of COVID-19 disease/vaccine will assist in preventing vision loss and oculomotor deficits.

### Financial Support and Sponsorship 

This article is authors' own work and has no sources of support.

### Conflicts of Interest

The authors have no financial interest in the subject of this article.
